# Hypothalamic Mitochondrial Dysfunction as a Target in Obesity and Metabolic Disease

**DOI:** 10.3389/fendo.2018.00283

**Published:** 2018-05-31

**Authors:** Juan Cunarro, Sabela Casado, Javier Lugilde, Sulay Tovar

**Affiliations:** ^1^Departamento de Fisioloxía and Centro de Investigación en Medicina Molecular (CIMUS), Universidade de Santiago de Compostela, Instituto de Investigaciones Sanitarias de Santiago de Compostela (IDIS), Santiago de Compostela, Spain; ^2^CIBER Fisiopatología de la Obesidad y Nutrición (CIBERobn), Madrid, Spain

**Keywords:** obesity, mitochondria, hypothalamus, treatment, glucose

## Abstract

Mitochondria are important organelles for the adaptation to energy demand that play a central role in bioenergetics metabolism. The mitochondrial architecture and mitochondrial machinery exhibits a high degree of adaptation in relation to nutrient availability. On the other hand, its disruption markedly affects energy homeostasis. The brain, more specifically the hypothalamus, is the main hub that controls energy homeostasis. Nevertheless, until now, almost all studies in relation to mitochondrial dysfunction and energy metabolism have focused in peripheral tissues like brown adipose tissue, muscle, and pancreas. In this review, we highlight the relevance of the hypothalamus and the influence on mitochondrial machinery in its function as well as its consequences in terms of alterations in both energy and metabolic homeostasis.

## Introduction

Maintenance of mitochondrial function across an individual’s life spam is needed for general homeostasis. In fact, one of the hallmarks of aging is alterations in mitochondrial function (1). In addition, mitochondrial dysfunction has emerged as a factor responsible for many metabolic diseases, such as obesity and type 2 diabetes (T2D) (2).

The mitochondrion is the principal organelle for the production of ATP in the cell. This essential process is localized in the inner membrane of mitochondria and involves the electron transport chain and oxidative phosphorylation. The generation of ATP could be mediated for different processes such as nutrient oxidation, autophagy, mitophagy, and apoptosis (3).

Mitochondria play an essential role for nutrient adaptation. In an environment with excess of nutrients, the energy storage is first used by the organism, and then the mitochondria play a key role because of its implication in bioenergetics and metabolism of amino acids and lipids (4). In this aspect, the bioenergetic implications of mitochondria were studied using different approaches in relation to energy demand and supply balance, resulting in a link with mitochondrial architecture (4). Specifically, it was demonstrated that a nutrient-rich environment is associated with a fragmented mitochondrial network, whereas fasting states is associated with elongated mitochondria (fusion machinery) (5, 6). In summary, the regulation of mitochondrial fission and fusion is necessary for eliminating damaged materials or other components in order to maintain the mitochondrial population in good conditions.

Neurons are cells with high energy needs, and therefore, the mitochondrial machinery plays an important role that ensures a constant energy supply and subsequently an adequate function of these cells (7). In addition and in relation to different nutrients, it was demonstrated that in lipid nutrient brain sensing, mitochondrial activity is implicated with the reactive oxygen species (ROS) signaling pathway (8). Nevertheless, although the regulation of energy homeostasis in hypothalamic neurons by mitochondria is very important, only few studies have addressed this issue, and we will focus on it in this review.

## Central Control of Energy Homeostasis

The regulation of energy homeostasis is essential for the maintenance of life. In this context, the central nervous system, and more specifically the brain, is the “master” that controls the regulation of food intake and energy expenditure through different neuronal circuits distributed to different areas in the brain (9).

It is generally accepted that the hypothalamus is a key region in the brain in the sense that it plays an essential role in modulating feeding behavior and energy expenditure. To this end, the hypothalamus integrates signals from the periphery and, through energy and nutrient sensing mechanisms, it generates an appropriate response by different organs (10, 11). From an anatomic-functional point of view, the hypothalamus is comprised of different nuclei, including the arcuate nucleus (ARC), paraventricular nucleus, lateral area (LHA), dorsomedial nucleus (DMH), and ventromedial nucleus (VMH) (12), all of which have an important role in energy homeostasis.

**Figure 1:**
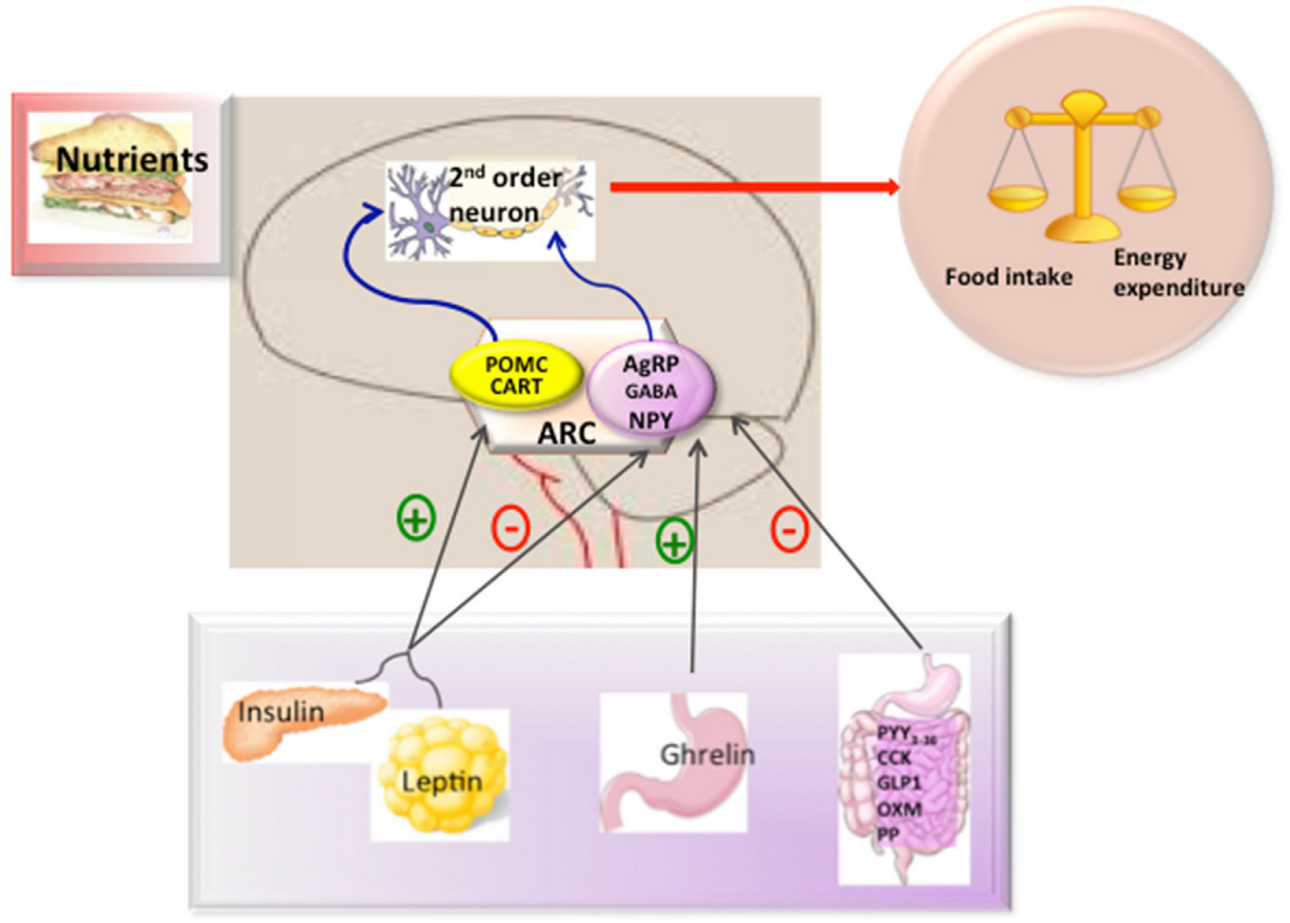
Hypothalamic control of energy metabolism. The brain integrates metabolic signals (leptin, insulin, ghrelin, PYY_3–36_) from peripheral tissues such as pancreas, adipose tissue, and gut. In the brain, specialized neuronal networks coordinate adaptive changes in food intake and energy expenditure. Neuropeptide Y/agouti-related protein (AgRP)-gamma aminobutyric acid (GABA) and proopiomelanocortin (POMC)-Cocaine and amphetamine-related transcript (CART) producing neurons in the hypothalamic arcuate nucleus primarily sense the body energy state. These neurons project to other second order neurons to control the regulation of energy balance. Abbreviations: ARC, arcuate nucleus; PYY3-36, peptide YY3-36; CK, cholecystokinin; GLP-1, glucagon-like peptide-1; OXM, oxyntomoduline; PP, pancreatic polypeptide.

In the ARC, there are two main neuronal populations that are involved in the regulation of energy balance and glucose homeostasis; one that co-expresses the orexigenic neuropeptides NPY (Neuropeptide Y), agouti-related protein (AgRP), and gamma aminobutyric acid (GABA) (13–15) and another that expresses amphetamine-regulator transcriptor (CART) and the anorexigenic neuropeptide α-MSH which is a processing product of the precursor proopiomelanocortin (POMC). These neurons could respond to peripheral signals from the adipose tissue (such as leptin), pancreas (such as insulin), and gut hormones [such as, ghrelin, glucagon-like peptide-1, peptide YY3-36, cholecystokinin (CK), and pancreatic polypeptide (PP)] (Figure 1) to maintain energy balance (16). In general terms, the effects of these peripheral signals are mediated by influencing the expression of the orexigenic neuropeptides NPY and AgRP or anorexigenic neuropeptides such as α-MSH acting through melanocortin receptors (17, 18).

Among the peripheral signals, there is little doubt about the important role played by leptin. The main effect of leptin in energy homeostasis is exerted in the hypothalamus. Leptin binding to its receptor (LepR-b) activates a signaling cascade, which leads to the activation of STAT3 and subsequent depolarization of POMC neurons, as well as hyperpolarization of NPY/AgRP/GABA neurons, thus, exerting its powerful anorexigenic effect (19, 20). Similarly, insulin also inhibits food intake, albeit to a much lesser extent than leptin, by binding to the central InsR. Upon this, IRS is activated through a classic phosphorylation cascade. The leptin signaling and insulin signaling converge in a common signaling pathway through PIP3K-PIP_3_-PDK1-FoxO1 (21). Within the nucleus, FoxO1 can activate AgRP and inhibit POMC expression (22). The activation of AKT for PDK1 produces phosphorylation of FoxO1 and leads to its translocation, facilitating STAT3 to bind to POMC/AgRP (22, 23).

In contrast to leptin, circulating ghrelin levels are increased in conditions of negative energy balance. Orexigenic effects of ghrelin are mediated by enhancing NPY and AgRP transcript expression though a mechanism which includes AMPK, p53, Sirt1, pCREB, FoxO1, and Bsx (24–26), as well as producing depolarization of POMC neurons, thus reducing α-MSH release (27–30).

The hypothalamus can also interact synergistically with other gut hormones, such as PYY3-36, CK, GLP-2, oxyntomodulin, and PP, to suppress appetite in contrast to ghrelin (11, 16) in terms of interaction with NPY/AgRP and POMC/CART.

## Mitochondrial Dynamics

**Figure 2:**
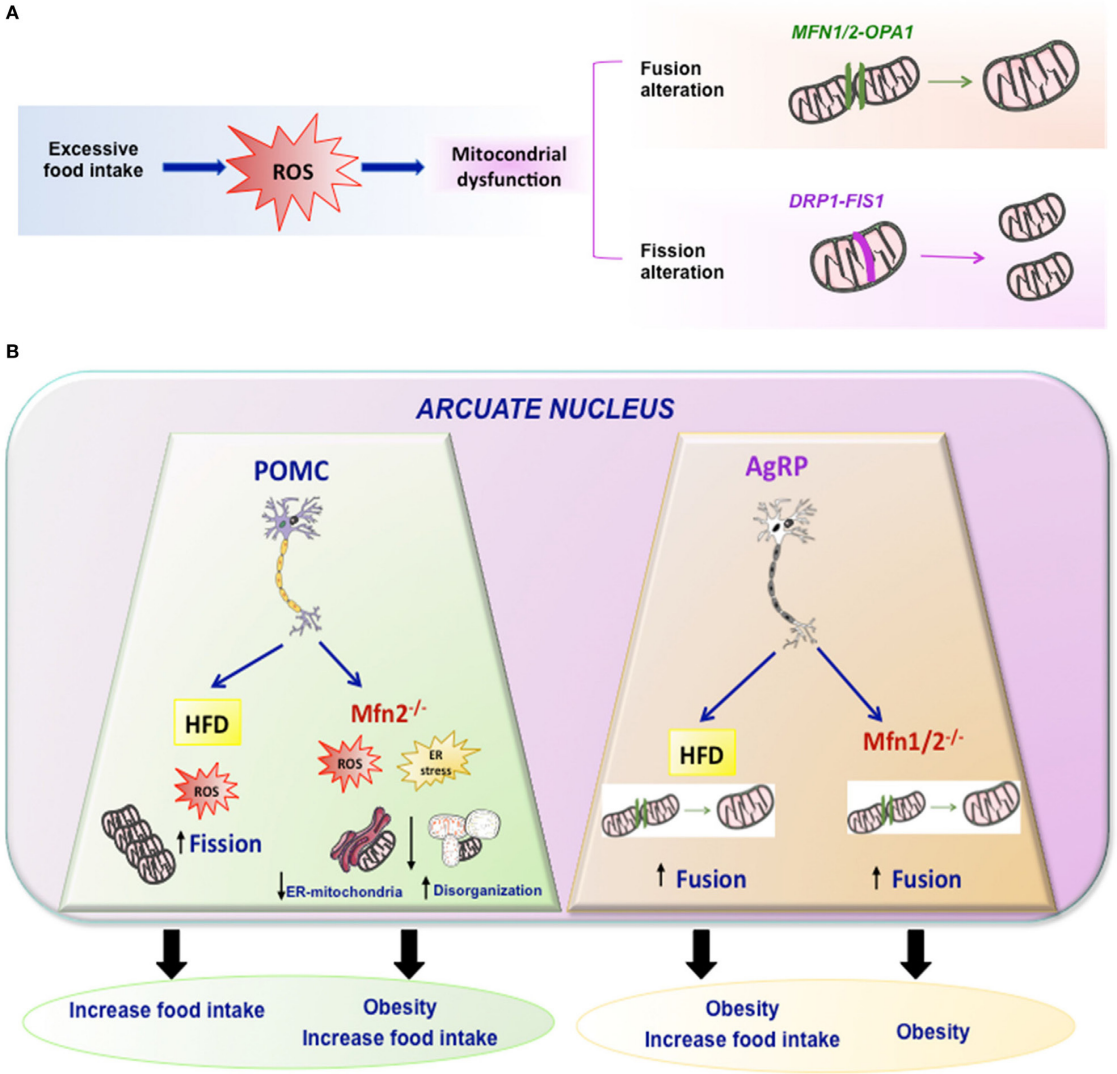
Mitochondrial dynamics. **(A)** Excessive food intake produce excess of ROS and mitochondrial dynamic dysfunction leading to altered fission or fusion. Fusion is driven by Mfn1 and Mfn2 localized on mitochondrial outer membranes, while OPA1 mediates mitochondrial inner membrane fusion. Mitochondrial fission is driven by DRP1 and it may involve FIS1. **(B)** Summary of the effects of HFD or mutations of Mfn2 or Mfn1/2 in specific arcuate neurons such POMC or AgRP in mitochondrial dynamic and energy homeostasis.

The combination of different events related to mitochondrial movements, such as fusion, fission, mitophagy, and tethering, are responsible for the mitochondrial architecture. In addition, the structural relation and functional contacts of mitochondria with other subcellular organelles is very important for regulation of energy metabolism. One well-known example is the association between ER and mitochondria in the ER–mitochondria-associated membranes (Figure 2) (31, 32). They are very important for the regulation of mitochondrial and cellular functions like mitochondrial division, apoptosis, or lipid and Ca^2+^ biogenesis (33, 34).

### Mitochondrial Fusion Machinery

Mitofusins (Mfn1 and Mfn2), GTP proteins localized in the outer membrane, as well as Optic atrophy (OPA1) localized in the inner membrane (35, 36) are the principal proteins involved in mitochondria compartmentalization for the mitochondrial fusion (37).

Mfn1 and Mfn2 are fusogenic proteins belonging to the family of transmembrane GTPases (38, 39). They are integral outer mitochondrial membrane proteins that require GTPase activity to mediate mitochondrial fusion. The function of Mfn1 and Mfn 2 seems to overlap, since Mfn1 partially rescues the defects caused by Mfn2 mutations (40, 41). Mitofusins are essential for mitochondrial fusion as shown by a study of *in vivo* KO models. Both Mfn1 and Mfn2 KO mice died in the middle of the gestation period (35), as a consequence of mitochondrial fusion failure in the placenta.

The protein or mRNA expression of Mfn1 and Mfn2 is variable among different tissues; Mfn1 is preferentially expressed in heart, liver, pancreas, adrenal glands, and testis (42, 43), whereas Mfn2 is more abundant in heart, skeletal muscle, brain, adrenal glands, and brown adipose tissue (4, 42, 44).

The mitofusin proteins regulate mitochondrial fusion, albeit at different stages. Indeed Mfn1 exhibits more efficiency in the mediation of mitochondrial docking and fusion than Mfn2. Moreover, Mfn1 is required to mediate Opa-1-driven mitochondrial fusion, showing both transcriptional and posttranscriptional regulation (43). Mfn2 exerts a key role in the brain, protecting against neurodegeneration in different brain regions, such as the cerebellum, hippocampus, and cortex (45), and in different populations such as dopaminergic neurons (46, 47).

In the periphery, Mfn2 promotes insulin signaling increasing insulin sensitivity since genetic ablation in muscle or liver resulted in impaired insulin signaling (48, 49). Furthermore, Mfn2 is involved in the association between ER and mitochondria (38).

As we have described, Mfn1 and Mfn2 have non-redundant and distinct roles. This could be partially explained by interactions with specific partners, modulators of their activity, and possibly distinct posttranslational regulations (4).

### Mitochondrial Fission Machinery

The fragmentation of a mitochondrion into two is a process called mitochondrial fission. Failure in this process elicits an increase in the generation of ROS and causes a mitochondrial population with non-uniform DNA distribution (50, 51).

The dynamic regulated protein 1 (DRP1) and FIS1 are involved in the regulation of mitochondrial fission. DRP1 is a cytosolic protein that is recruited to the outer mitochondrial membrane where the scission takes place. Once DRP1 assembles into mitochondrial fission sites, it produces the hydrolysis of GTP thereby promoting its division (52–54). Interestingly, DRP1 is regulated by posttranscriptional modifications like phosphorylation and SUMOylation (55). In addition, DRP1-ablation causes embryonic lethality in mice, whereby in embryos there appeared alterations in the liver, alterations in heart development, and deficient synapse formation (56). On the other hand, FIS1 is located mainly in the OMN, and it is linked *via* its COOH terminal part to the outer mitochondrial membrane. Studies investigating mutations showed that an overexpression of FIS1 produced mitochondrial fragmentation, and knock down of this protein caused a highly fused mitochondrial network, indicating that FIS1 activates mitochondrial fission (57–59). Finally, MFF (mitochondrial fission factor), MiD49, and MiD51 are other mitochondrial fission proteins that also participate in this process (60, 61).

## Hypothalamic Mitochondria and Regulation of Energy Metabolism

It has been recognized for some time that mitochondrial dysfunction is at the root of some of the pathophysiological features related to metabolic disorders such as diabetes. Thus, it was shown some time ago that the “metabolic inflexibility” linked states of insulin resistance in T2D can be explained to some extent by alteration in mitochondrial activity in peripheral tissues such as muscle (5, 62). Although the presence of mitochondrial dysfunction in T2D is beyond doubt (63), whether this is a cause or a consequence and whether improvement/deterioration in insulin sensitivity run in parallel to changes in mitochondria dynamics is controversial.

In addition, in the liver, mitochondrial dynamics have an important role in the fasting–feeding transition and maintaining postprandial mitochondrial quality; and it is directly connected to some clock genes such as diurnal Bmal1 gene (64).

Mitochondria participate in many processes and mitochondrial dysfunction is thought to trigger many chronic diseases, including liver disorders, diabetes, and aging processes (65, 66). In addition, mitochondria is very important for the development of obesity because it integrates different metabolic information, such as ATP levels, oxidative stress, ER stress, inflammation, and cell signaling, which play important roles in the onset and maintenance of obesity.

Several studies have linked obesity with mitochondrial alterations (67–70). This is because the excessive intake of nutrients provokes the mitochondria to become overloaded with fatty acids and glucose, leading to an increase in the production of Acetyl-CoA. This then causes the production of NADH through the Krebs cycle, which promotes an increase of electrons with the ability to enter the mitochondrial intermembrane space, which subsequently produces an excess of ROS, leading to *oxidative stress* to the cell (71, 72). This causes the activation of several transcription factors, among which is NFκB, the main mediator of the inflammatory response (73).

Oxidative stress has been associated with adiposity, insulin resistance, and metabolic syndrome, and it is postulated that oxidative stress could be an early event in the etiology of these chronic diseases (74, 75).

Several studies have shown using murine models the relevance of these phenomena in obesity. Ma and collaborators identified that diet-induced obesity in rats produced an increase of oxidative stress and mitochondrial dysfunction in the brain; they also showed that excessive fat intake reduced the activity of the main antioxidant enzymes (SOD, CAT, GPx) (76). More specifically within the hypothalamus, the neuronal activity of both POMC neuron and AgRP neuron populations is modified by ROS (18, 77, 78). More recently, it was demonstrated that the link between ROS and effect of food intake in POMC neurons could be mediated by mTORC (79).

Another phenomenon connected with obesity development is *ER stress*. ER stress is produced by the imbalance between protein folding capacity and protein load (80). The direct result of ER stress is the accumulation of misfolded proteins. The association of obesity and ER stress is essential for mitochondria and RE, and it is modulated at least partially by mitochondrial fission and fusion (81).

The mitochondrial dysfunction is a result of decreased ATP production for ER stress and alterations in mitochondrial membrane, and it has been described that diet-induced obesity and genetics factors in mice are associated with increased ER stress in the hypothalamus (82, 83). In this aspect, Mfn2 has emerged as an important link between mitochondria and ER. In fact, its ablation has been found to be associated with ER stress in different cell types and tissues (49, 84). Moreover, there is a multitude of evidence suggesting that hypothalamic ER stress is a causative factor in the development of obesity by inducing leptin and insulin resistance (85, 86). In addition, ER stress mediated the effects of thyroid hormones on energy balance (87) approach for ameliorating the genetic obesity exhibited by obese Zucker rats or diet-induced obesity in normal rats (88).

Taking into account that Mfn 2 plays a key role in mitochondrial fusion and establishing ER–mitochondria interactions, some studies generated KO mice with specific deletions of Mfn 2 or Mfn1 in specific hypothalamic neurons such as POMC or AgRP (89, 90). Animals with a specific deletion of Mfn 2 in POMC neurons exhibited a striking phenotype. The main features could be summarized as follows: POMCMfn2KO in the arcuate nucleus exhibited marked obesity, which was due to both increased food intake and reduced energy expenditure associated with decreased BAT activity and reduced expression of thermogenic markers (UCP-1) in BAT. These animals exhibited early onset leptin resistance and presented the expected alterations in mitochondrial morphology, mitochondria–ER contacts, intra-mitochondrial respiratory capacity, and enhanced ROS production, as well as ER stress. The marked phenotype presented by these animals was specific for Mfn2 since animals with deletion of Mfn1 did not show this phenotype and had normal body weight. Such marked phenotype was indeed due to ER stress since its inhibition by chemical chaperones, such as TUDCA, led to an almost complete reversal of the obesity-related phenotype of POMCMFN2KO mice (90). In addition, other study indicate that activation of CB1R receptors, that induces a well-known hyperphagic response, is associated to a paradoxical activation of POMC neurons and that such response involves β-endorphin release—but not α-msh—from these neurons, and a UCP2-dependent mitochondrial switch in these neurons leading to ROS production and changes in mitochondria–ER interaction (91).

The data described above by Schneeberger et al. (90) appear to be neuron-specific since a similar approach conducted by deleting Mfn1 or Mfn2 in AgRP neurons led to a very different phenotype (89). In fact, there was no evidence of ER stress in any of these animals in contrast to when the deletion was performed in POMC neurons. The most interesting findings in these mice were related to altered mitochondrial size and density. In terms of energy balance, the most striking feature was that these animals were resistant to HFD-induced obesity. Although the mechanism is not clear-cut, this could be largely due to decreased ATP bioavailability, which led to impaired electrical activity in these neurons (89).

Independent of the fact that these results indicate the existence of different mechanisms in both neuronal cell types, they demonstrate that the whole body response to HFD is markedly dependent on specific patterns of mitochondrial dynamics in these neurons.

## Mitochondria Brain Dysfunction and Impaired Glucose Metabolism

The evidence regarding the relevance of mitochondrial function in glucose homeostasis is extensive. Among the supporting evidence includes data showing changes in mitochondrial structure in postmortem islets derived from patients with diabetes (92) and data showing that mutations in mitochondrial DNA (93) can be linked to higher incidence of glucose intolerance. Further, a more direct support came from experimental studies showing that depletion of mitochondrial DNA in INS-1 cells led to an impairment in glucose-induced mitochondrial membrane potential hyperpolarization and insulin secretion (94).

Finally, recent studies demonstrated that obese Zucker rats with impaired glucose sensing in the hypothalamus showed an abnormal redox signaling that originates from the mitochondrial function (95); nevertheless, when the redox state was restored, the glucose sensing in the brain was also restored (96).

Although such data provided conclusive evidence of the relevance of mitochondrial dysfunction in relation with impaired glucose metabolism, recent evidence uncovered a further mechanism acting at the hypothalamic level. Following data obtained with selective deletion of Mfn2 in energy homeostasis, some authors undertook a similar approach with Mfn1 in relation to glucose homeostasis. Selective deletion of Mfn1 in POMC neurons led to structural and functional alterations in mitochondria (90). These changes led to abnormal glucose homeostasis, which was due to impaired pancreatic insulin secretion. A more recent publication from this group also demonstrated other consequences in the alteration of Mfn1 in mitochondrial dynamics, which are essential for the appropriate responsiveness of POMC neurons to the increase or decrease of glucose concentrations (97). Nevertheless, not only the proteins implicated in fusion mechanism but also mitochondrial fission regulators like DRP1 were directly implicated in the control of glucose sensing in POMC neurons (98). In addition, data from ventromedial hypothalamus neurons clearly demonstrated that increased extracellular glucose concentrations resulted in UCP2-dependent mitochondrial fission in these neurons and this effect was mediated by DRP1 (dynamin-related peptide-1) (99). This unsuspected finding led to the hypothesis of considering mitochondrial remodeling in these hypothalamic neurons as a sensing mechanism implicated in whole body homeostasis.

More studies confirmed the importance of astrocytes in the co-regulation of hypothalamic glucose sensing and systemic glucose metabolism. In relation with mitochondrial dynamics, the astrocyte-specific loss of insulin receptors produce alterations in mitochondrial integrity and mitochondria–ER contacts in POMC neurons, impairing the appropriate response of these neurons to glucose changes (100).

At to which point these neurons can be considered a target of current drugs for clinical use is currently being investigated. Peripheral treatment with metformin improved peripheral insulin resistance, decreased plasma and brain oxidative stress, and restored brain mitochondrial function, that were all produced from high-fat diet (101). On the other hand, direct administration of metformin in the brain for up to 7 days in mice caused severe hypoglycemia and alterations in mitochondrial function/viability (102). Finally, using an experimental model of diet-induced obesity in rats, it was found that HFD caused brain mitochondrial dysfunction indicated by an increase of brain mitochondrial ROS and increase of the size of mitochondria in the brain (103). In this model, using incretin-drug-based vildagliptin in obese insulin-resistant rats ameliorated peripheral insulin resistance and improved brain insulin sensitivity and mitochondrial function (104, 105). Dapagliflozin, a sodium glucose cotransporter 2 (SGLT2) inhibitor, also restored peripheral insulin sensitivity and decreased brain oxidative stress, which led to improved brain mitochondrial function, brain inflammation, normalized brain insulin signaling, improved brain insulin sensitivity, decreased brain apoptosis, and increased hippocampal synaptic plasticity (106). Further studies assessing the effects of direct administration in specific hypothalamic nuclei and neuronal populations are needed in order to clarify which specific neurons are involved in these effects.

## The Mitochondrial Bioenergetics in the Hypothalamus as New Therapeutic Target

Data gleaned in recent years suggest that mitochondrial bioenergetics could represent a useful target in different diseases spanning the fields of cancer, metabolism, or neurodegeneration to mention a few (1, 90, 107–110).

Overall, currently available data strongly document that mitochondrial fusion proteins are novel pharmacological targets (111, 112). This potential is related to the major impact that mitochondria plays in cell bioenergetics since they are the most relevant producer of ROS as well as the main antioxidant (113), both of which are involved in the root and/or progression of many diseases. However, it has become a daunting task to develop specific compounds, without side effects, that could be targeted to a specific cell population affected by the disease and not healthy cells (114). In order to overcome such pharmacological issues, some studies have tried to develop alternative strategies linked to changes in lifestyle or nutritional intake, such as caloric restriction, physical exercise, or diet supplementation with antioxidants (115–117). Although there are clear benefits in disease progression in some instances with these approaches, it remains unclear as to what extent this process is mediated by changes in mitochondrial bioenergetics. Another indirect approach is to try to influence the activity of targets that could in turn influence mitochondrial function. Among these approaches are studies assessing the influence of rosiglitazone, resveratrol, or metformin (118, 119), which could exert their effects *via* PPAR, Sirtuins, or AMPK. However, it is also unclear as to what extent the effects of the aforementioned targets are dependent on mitochondrial function, since these drugs can exert their action independently of these targets. In any case, it should be emphasized that most studies failed to document a clear-cut benefit in those patients with severe mitochondria dysfunction.

A more direct strategy would be to target the cell machinery directly involved in mitochondrial biogenesis and/or function. One example of this is the search for molecules that upregulate Mfn2 expression and induce mitochondrial fusion (111) led to discovery of a molecule, called leflunomide, that was approved by the FDA (111). This compound induced the expression of Mfn2 and Mfn1 in C2C12 muscle and in HeLa cells (120). The active metabolite for leflunomide is teriflunomide, which also increases Mfn2 transcriptional activity and mitofusin mRNA levels in Hela cells. Interestingly, it was found that leflunomide inhibits dihydroorotate dehydrogenase (DHODH), an inner mitochondrial membrane enzyme that catalyzes the fourth step in the novo synthesis of pyrimidines, *in vitro* (121, 122). Additional experiments showed that leflunomide and teriflunomide induced the upregulation of mitofusin and mitochondrial elongation by depletion of the cellular pyrimidine pool secondary to DHODH inhibition (123). This study also demonstrated that deficiency in cellular pyrimidine pool induced mitochondrial fusion using BRQ (brequinar) and DHODH inhibitor. Using different experiments with inhibitors of the components to different pathways, the authors showed that Leflunomide blocks pyrimidine biosynthesis by inhibiting DHODH and thus functionally connected with respiratory complex III. In addition, other inhibitors of Complex III or DHODH also increased mitofusin expression and promoted mitochondrial fusion (111). All these studies may lead to new strategies for the treatment of patients with genetic defects who exhibit marked alterations in mitochondrial dynamics, and for which there is currently no adequate treatment. At present, the major hope is focused in the development of gene therapy or exogenous stem cell treatments for which preclinical studies support their feasibility, although some issues still need to be resolved before being applied in the clinical setting (112).

## Concluding Remarks and Perspectives

Mitochondrial dynamics is essential for maintaining cell homeostasis and their dysregulation has been historically linked to different rare diseases which usually share common features of patients suffering marked disabilities. Most recently, they were also linked to some high incidence diseases such as neurodegenerative diseases, cancer, and diabesity (obesity and T2D). From a basic point of view, several seminal discoveries were made over the last few years. These include the characterization of the molecular machinery involved in mitochondrial dynamics and their mechanism of action, in terms of cell bioenergetics and maintenance of adequate control of ROS. In addition, the generation of *in vivo* models for these molecules using standard gain/loss of function approaches in specific neuronal cell populations helped to uncover some unsuspected biological roles. This led in some instances, e.g., mitofusins, to be now considered as major regulators of energy homeostasis at the whole body level. Even more, Mitifusin 1 has emerged as a nutrient sensor in hypothalamic (POMC) neurons that plays a key role in the central control of insulin release from the pancreas. In fact, these components of the mitochondrial machinery are being considered as drug targets for diabesity despite the difficulties related to this approach. Nevertheless, taking into account the well-known interrelationship between diabesity, cancer, and neurodegenerative disease, and the sharing of some common molecular features that are involved with mitochondrial bioenergetics at the root of these comorbidities is being studied in depth. Overall, these major breakthroughs should translate into new therapies in the near future. While most attempts thus far have been linked to failure or at most no clear benefit, new approaches like gene therapy may overturn this current status. Such therapies are something desperately needed, since there is none currently available for many of the patients with severe genetic defects in mitochondrial function.

## Author Contributions

JC, SC, and JL contributed with the elaboration of the manuscript and bibliography searching. ST elaborated the manuscript and made the final revision.

## Conflict of Interest Statement

The authors declare that the research was conducted in the absence of any commercial or financial relationships that could be construed as a potential conflict of interest.
